# Airway Clearance Techniques: The Right Choice for the Right Patient

**DOI:** 10.3389/fmed.2021.544826

**Published:** 2021-02-04

**Authors:** Stefano Belli, Ilaria Prince, Gloria Savio, Elena Paracchini, Davide Cattaneo, Manuela Bianchi, Francesca Masocco, Maria Teresa Bellanti, Bruno Balbi

**Affiliations:** Pulmonary Rehabilitation Department, Istituti Clinici Scientifici Maugeri, Istituto di Ricerca e Cura a Carattere Scientifico (IRCCS), Institute of Veruno, Novara, Italy

**Keywords:** airway clearance techniques, disability, rehabilitation, COPD, bronchiectasis, amyotrophic lateral sclerosis, positive pressure, negative pressure

## Abstract

The management of bronchial secretions is one of the main problems encountered in a wide spectrum of medical conditions ranging from respiratory disorders, neuromuscular disorders and patients undergoing either thoracic or abdominal surgery. The purpose of this review is illustrate to the reader the different ACTs currently available and the related evidence present in literature. Alongside methods with a strong background behind as postural drainage, manual techniques or PEP systems, the current orientation is increasingly aimed at devices that can mobilize and / or remove secretions. Cough Assist, Vacuum Techniques, systems that modulate airflow have more and more scientific evidence. Different principles combination is a new field of investigation that goes toward an increasing of clinical complexity that will facing us.

## Introduction

The management of bronchial secretions is one of the main problems encountered in a wide spectrum of medical conditions ranging from respiratory disorders (e.g., COPD, bronchiectasis, cystic fibrosis) to neuromuscular disorders (e.g., ALS) to patients undergoing either thoracic or abdominal surgery. The term airway clearance techniques (ACTs) refers to a variety of different strategies used to eliminate excess secretions. Their aim is to reduce airway obstruction caused by secretions occupying the airway lumen and so prevent respiratory tract infections, re-expand the collapsed areas of the lung, thus improving gas exchanges and decreasing the inflammatory response (1–4).

A wide range of treatments, techniques and devices are present in the scientific literature for managing bronchial encumbrance in respiratory physiotherapy; but their very multiplicity begs the question in daily clinical practice: “which of these treatments is it better to use to obtain the better result in my patient?.” Up to now, for all ACTs there is insufficient evidence to prove their efficacy and effectiveness in different clinical scenarios or to affirm the superiority of one technique over another (5–8). This absence of evidence, however, does not necessarily mean an absence of efficacy. Obviously, there is a need for more studies to increase the body of scientific evidence on this topic (9).

Looking at the issue from the other side, i.e., from the patient's perspective, rarely is just one single technique used for a given pathological condition. In addition, for many patients and/or categories of patients the goal might be to combine the best effect on clearance of the airways with the lowest possible incidence of side effects and of adverse events, such as exacerbation of the underlying pathophysiology (10). According to Lapin (11), the overall effectiveness of any technique is influenced by several factors closely related to the patient. Adherence to treatment is fundamental and it depends very much on the patient's satisfaction, motivation and perceived effectiveness. Therefore, it is essential always to take into account the patient's preferences, and base one's choices about which technique to use not only on the relief of symptoms, but also on the adaptability of the technique to the patient's lifestyle.

Underlying these numerous techniques, however, are a series of different physiological mechanisms used for “unblocking” the obstruction:

- Increase of expiratory flow
- Oscillation of the airflow
- Increase in lung volumes.

**Figure 1:**
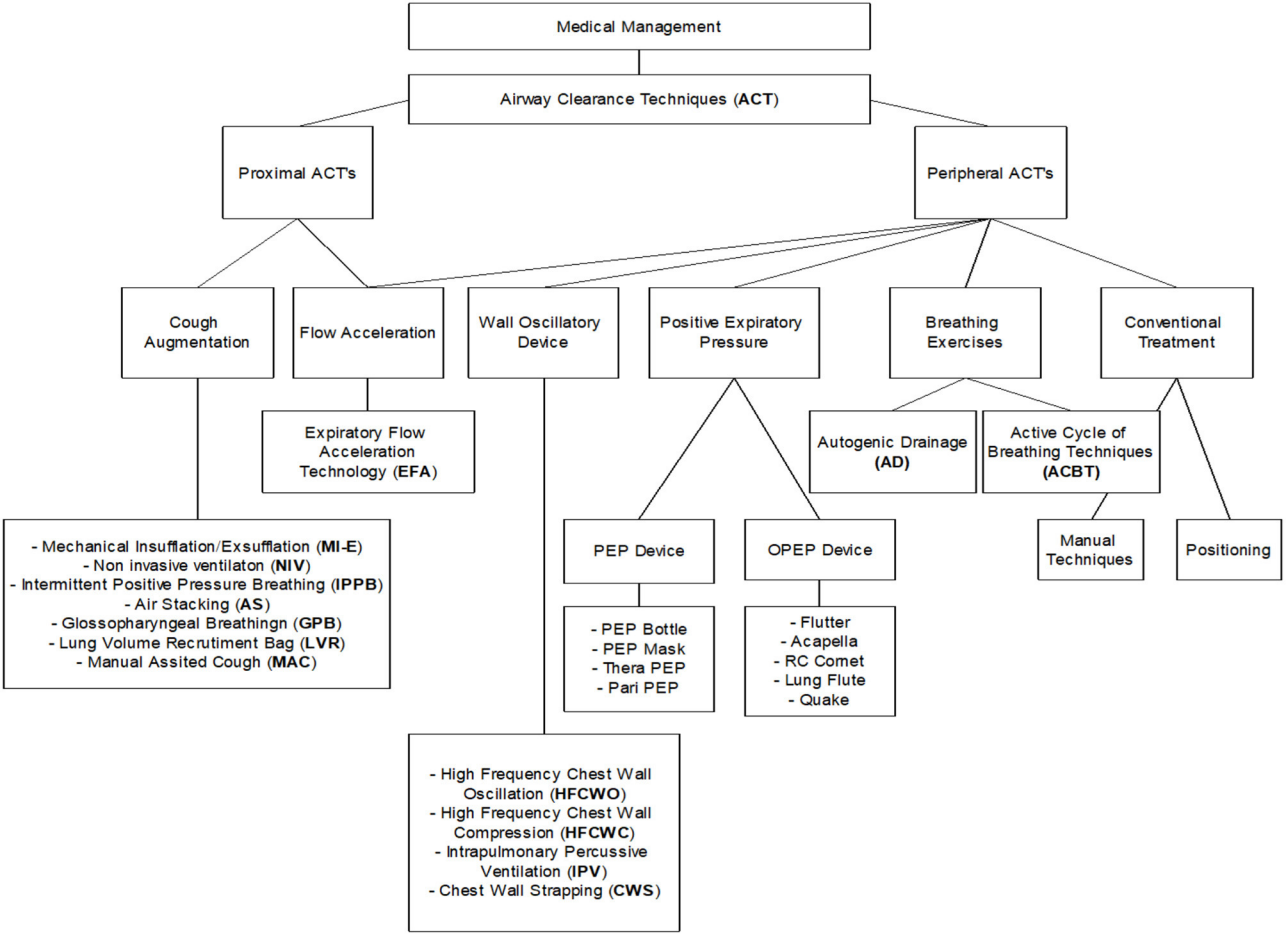
A schematic representation of a proposed algorythm for different ACTs.

The aim of this review is to illustrate to the reader the different ACTs that are currently available (Table 1 and Figure 1). Although our paper is not a systematic review nor a meta-analysis, we hope it can help the reader gain a better understanding on how to proceed when choosing ACTs for their patients.

**Table 1:** Different ACTs and their characteristics.

	**Patients typology**	**Degree of collaboration of the patient**	**Cough efficacy**	**Pro**	**Con**	**Time of execution**	**Need for …**
Postural Drainage	COPD, cystic fibrosis, post-surgery (precaution)	Average - Good	Yes	Low cost, studied for a long time, combine with other techniques	Patients with reduced flows, patients with cognitive impairment, highly deconditioned patients, patients with reduced cough reflex, thoracic trauma	10′−30′ (2–3 times/day)	Mobilize secretions from periphery of the lung
Manual techniques	COPD, cystic fibrosis	Average - Good	Yes	Low cost, detach secretions from the bronchial wall, Combine with other techniques	Patients with reduced flows, patients with cognitive impairment, highly deconditioned patients, patients with reduced cough reflex	20′ (2–3 times/day)	Mobilize secretions from periphery of the lung. Facilitate expectoration
Active Cycle of Breathing Techniques	COPD hypersecretive Bronchiectasis Cystic fibrosis Pre/post-surgery	Good	Yes	Free. It combines lung re-expansion and PEP effect.Self-management	Patients with reduced flows, patients with cognitive impairment, agitated or confused, patients not spontaneously breathing	20′ (2–3 times/day)	Mobilize secretions from the periphery of the lung. Facilitate expectoration
Autogenous Drainage	COPD hypersecretive Bronchiectasis Cystic fibrosis Pre/post-surgery	Average - Good	Yes	Free. It combines lung re-expansion and PEP effect. Self-management	Patients too young, patients with cognitive impairment, highly deconditioned patients, patients with reduced cough reflex, difficult to teach	20′ (2–3 times/day)	Mobilize secretions from the periphery of the lung
PEP Systems (PEP mask, PEP Bottle, thera PEP. etc.)	COPD hypersecretive Bronchiectasis Cystic fibrosis Pre/post-surgery	Good	Yes	Low cost, easy to use and transportable. Excellent for managing the early stages of COPD. Self-management	Patients with reduced flows, patients with cognitive impairment, highly deconditioned patients, Pneumothorax, active haemoptysis	10′−15′ (1–2 times /day)	Mobilize secretions from the periphery of the lung, increasing lung volume (FRC and VT), reduction of hyperinflation
OPEP Systems (Aerobika, Acapella, Flutter, Cornet, etc.)	COPD hypersecretive Bronchiectasis Cystic fibrosis Pre/post-surgery	Average - Good	Yes	Low cost, easy to use and transportable, the vibration allows a better action on the denser secretions. Excellent for managing the early stages of disease. Self-management.	Patients with reduced flows, patients with cognitive impairment, highly deconditioned patients, Pneumothorax, active haemoptysis	10′−15′ (1–2 times/day)	Mobilize secretions from the periphery of the lung; the swing facilitates the detachment of secretions, increasing lung volume (FRC and VT), reduction of hyperinflation
Cough Assist (E70, Kalos, Nippy, etc.)	NMD, post coma, ABI (with attention to glottic functionality)	From Good (in synchrony with device) to Absent	NO	Useful for those with: weak muscle, conditions with ineffective cough, In case of ineffectiveness of the air-stacking maneuver, Weakness of respiratory mm, with ineffective cough (Peak cough flow (PCEF) <270 l / min.) Maximum Expiratory Pressure (MEP) <50 cmH_2_O highly studied machines, guidelines present x NMD	Complete paralysis of the glottis (risk of collapse of the airways in exhalations), Recent barotrauma, pneumothorax, Hemodynamic instability, Recent thoracic surgery, Bullous emphysema, pneumomediastinum, Recent abdominal surgery, Maxillofacial trauma, Epistaxis, requires trained staff, Expensive	10′−30′ (2–3 times/day)	Mobilize and remove secretions in patient with ineffective cough reflex using a Δ pressure
IPPV (or percussionaire)	COPD (hypercapnic), bronchiectasis, Cystic Fibrosis	From Good to Low	Yes	Form of ventilation, it also helps to reduce hypercapnia and improves oxygenation; useful for patients with thick secretions.	Expensive, not widespread; to know its use well. Difficult to domicile	30′	Optimize the ventilation of the patient; facilitate the detachment and ascent of secretions (through the “rupture” of the mucus)
T -PEP (temporary positive expiratory pressure)	COPD hyper secretive Bronchiectasis Cystic fibrosis Pre/post-surgery	Average - Good	Yes	Good feedback for the patient during the execution of the maneuver. Useful for ACOS patients, patients with reduced expiratory flows. Self-management.	Patients with cognitive impairment, with reduced cough reflex. costly	15′−20′ (1–2 times/day)	Maintain a prolonged and constant expiratory flow with low pressures facilitating the ascent of mucus
Vest (o smart Vest)	COPD, Bronchiectasis, Cystic Fibrosis	From good to absent	Yes	Easy to use, comfortable	For patients with ineffective cough need to join a Cough assist; expensive	20′ /die (even several times a day)	Mobilize secretions through vibrations on the chest wall.
Expiratory Flow Acceleration	COPD, Bronchiectasis, post-surgery, post-transplant, Cystic Fibrosis, NMD (Healthy Lung), ABI (if impossible use cough assist)	From good to absent	Not necessarily	Cystic fibrosis Self-management. Pre/post-surgery (thoraco-abdominal, ENT, cardiac) Brain damage Neuromuscular pathologies (ALS, MS, etc.) Excellent for tracheostomized patients and with low flows Self-management even for the most compromised patients	Not effective on very dense and viscous secretions (need to act on the rheology of the mucus); ineffective on tachypnoeic patients (the patient must breathe on his TV)	15′−30′ (but more in monitored and hypersecreted patients)	Accelerate the expiratory flow (Venturi system) facilitating the ascent of the secretions up to the upper airways or to the glottis (to then be swallowed)
NIV	Cystic fibrosis, Pre/post-surgery	Average - Good	Yes	Amplifies inspired flow. It combines lung re-expansion and PEP effect.	For patients with ineffective cough need to join a Cough assist; expensive	20′ /die	Re-expand and unblock areas at risk of atelectasis or bronchial encumbrance

## Description of Different Airway Clearance Techniques

### Postural Drainage

Postural drainage (PD) was one of the first techniques used. Nelson (12) was the first to describe the use of precise postures based on the anatomy of the bronchial tree. This technique exploits the force of gravity in order to facilitate the sliding of the mucus from the periphery toward the central airways where, with coughing, forced expiration (FET) or bronchial aspiration, it can be removed.

There are conflicting opinions in the literature about this technique. Several studies have investigated PD using radioactive aerosol to mark mucus and evaluate its clearance during PD, but none of them has ever confirmed the assumption that gravity alone can promote mucus displacement. In 2002, an article in Respiratory Care (Fink JB) stated that gravity is not a physiological mechanism for transporting mucus but is important for lung function, i.e., it has an effect on ventilation, perfusion and lymphatic drainage. Other authors such as Cecins (13) and Eaton (14) showed that PD is more effective than oscillatory positive expiratory pressure (OPEP) or Active Cycle of Breathing Techniques (ACBT) used alone in terms of the volume of secretions eliminated. However, several patients find this technique uncomfortable, as it increases the sense of dyspnoea (15). Fink (16) stated that “…PD has been shown to have little or no effect in diseases associated with low content of secretions, thus the signs indicating PD are largely limited to patients who have secretion production >30 ml per day….” Some authors, such as Bott (17), suggest that if severely affected patients find PD helpful during exacerbations, concomitant use of Intermittent Positive Pressure Breathing (IPPB) or Non-Invasive Ventilation (NIV) might help overcome the sense of increased dyspnoea with the additional benefits of PEP therapy for secretion management.

### Manual Techniques

These are techniques that involve applying certain forces to the patient's chest using the hands. Among the best known and most used, we find:

- Percussion (or Clapping): a rhythmic succession of rapid and light strokes performed with cupped hands on the patient's chest wall. The technique is applied to the specific segment to be treated while the patient breathes at a tidal volume (therefore both during inspiration and during exhalation). Percussion strength should be based on patient feedback (it must not create discomfort). The frequency to use must be between 4.6 and 8.5 Hz (18).
- Vibration: the application, during the whole exhalation phase, of fine oscillatory movements combined with a compression of the chest wall. The force that the therapist uses must be enough to compress the rib cage and increase the expiratory flow but, at the same time, it must not create discomfort for the patient.

There is some evidence regarding the physiological effects of these manual techniques on the clearance of secretions. During these techniques, an intermittent positive pressure is applied on the chest wall; this pressure is then transmitted to the airways causing an oscillation of the airflow and an increase in expiratory flow (18), i.e., two of the three afore-mentioned physiological mechanisms that should help clear the airways. However, to date there is no strong evidence to support or reject the use of manual techniques compared to other clearance interventions (5, 19). Some studies show that a combination of PD and percussion or vibrations are equally useful to other unblocking techniques as clearance strategies (6), improving sputum production when used in addition to forced expiration (FET) and PD techniques (20). McCarren and Alison (18) made a comparison between the OPEP devices (Flutter and Acapella) and manual techniques and showed that, although the OPEP devices produced higher oscillation frequencies than vibrations and percussions, the vibrations produced higher expiratory flow rates. So long as there is no high-quality research giving us a definitive answer on the effectiveness of these interventions, it is necessary to depend on the patient's preference. It can therefore be said that manual techniques are useful in patients who prefer these techniques or who are unable to collaborate in the treatment (patients with neuromuscular weakness, with cognitive problems, unconscious, strongly sedated, too young, etc.) as they are manual techniques, administered by an operator (physiotherapist or caregiver). However, it is important to emphasize that the evidence suggests that patient adherence to treatment is greater if self-administered techniques are used (17).

### Respiratory Techniques

#### Active Cycle of Breathing Techniques

In 1979, Pryor et al. (21) described a clearance strategy characterized by the fact that it does not require the use of specific equipment, for which reason it is preferred by many patients. The strategy is called Active Cycle of Breathing Techniques (ACBTs) and consists of three distinct breathing cycles performed in sequence: Breath Control, Thoracic Expansion Exercises (TEE) and Forced Exhalation Technique (FET). Breath Control simply consists of breathing at tidal volume, using a diaphragmatic breathing pattern, at the patient's own frequency and respiratory volume. This allows recovery from fatigue, desaturation, signs of bronchospasm and possible dyspnoea that may have occurred during the most active components of the cycle (22). The TEE consist of three or four respiratory acts characterized by a slow, deep inhalation (larger volumes than the tidal volume) through the nose, with a pause of about 3 s at the end of inspiration, followed by a passive exhalation. This deep inhalation should facilitate the collateral ventilation, and then the flow of air through the intrabronchial channels of Martin, the bronchoalveolar channels of Lambert and the interalveolar pores of Kohn (23). In this way, the patient should be able to bring air behind the secretions and then expand those areas that are blocked. Finally, the patient has to perform the FET, which consists of a combination of one or two forced expirations, called “Huff” and a tidal volume respiratory act. This exercise is most effective when the length of the Huff and the contraction force of the expiratory muscles determines the maximum expiratory airflow, minimizing the collapsing the airways. Usually, ACBT is effective when applied in a vertical position, but in reality it can be combined with other devices and/or in other positions (13) or in conjunction with manual techniques (percussion or vibrations). A systematic review of Cochrane compared the clinical efficacy of ACBT with other clearance strategies, and concluded that there is not enough evidence to suggest that ACBT is superior to any other technique. One can consider it comparable to other techniques in terms of patient preference, lung function, sputum weight, oxygen saturation and number of lung exacerbations (24), and comparable in terms of lung function, exercise capacity and quality of life for a long-term use (25).

#### Autogenous Drainage

Another clearance strategy that requires no specific device or equipment is Autogenous Drainage (AD). Developed in the 60s in Belgium, AD is characterized by three phases: “Unstick,” “Collect,” and “Evacuate” (26). Each phase consists of a series of respiratory acts in which a certain lung volume is mobilized (the volume mobilized in the “Evacuate” phase will be greater than that in the “Collect” phase which in turn will be greater than that mobilized in the “Unstick” phase). The rationale behind this technique is that shear forces are generated, through the exhaled airflow to the various lung volumes, which should reduce the adhesion of the mucus, detach the secretions from the bronchial walls and transport them from the peripheral airways to the proximal ones (26). It is important to underline that this strategy, at a technical level, is very complex to perform and therefore it may be difficult for some patients. Several studies have compared the AD technique to other clearance methods. These include the study conducted by Pryor (25) which demonstrated its clinical equivalence, in terms of all measured results, including quality of life and lung function, with the other methods analyzed (AD, ACBT, PEP, Flutter and RC-Cornet).

#### PEP Mechanisms

In the late 1970s, the use of Positive Expiratory Pressure (PEP) as an airway clearance strategy, introduced in Denmark, became widespread. PEP therapy consists in exhaling against a flow or a resistance at the threshold in order to produce positive pressure in the airways throughout the expiratory phase. The rationale behind this therapy is that it promotes, during inspiration, the flow of air beyond the obstruction through the collateral channels, causing the accumulation of a greater volume of air behind the secretions. This should create a pressure gradient astride the obstruction, which would favor the movement of the secretions in a centripetal direction (27, 28). Furthermore, during exhalation, the positive pressure generated prevents premature collapse of the peripheral airways (29). The PEP therapy most used in clinical practice is the low-pressure type (also referred to as “low PEP”). It consists in maintaining a relatively low expiratory pressure in the mouth, between 10 and 20 cmH_2_O. Inspiration is required for a slightly larger volume of air than the tidal volume, with a pause at the end of inspiration (to allow the physiological mechanisms of Pendelluft flow, interdependence and collateral ventilation) and then a slightly active exhalation against resistance. A variant of PEP therapy, used much less frequently in clinical practice, is high-pressure PEP (“HiPEP”) (30). It is used especially in patients who have an airway instability during forced expiration. Pressures ranging from 40 to 120 cmH_2_O are used, thus allowing to perform a forced expiratory maneuver (FET). The application then of a PEP during a FET is to avoid the premature collapse of the airway during the maneuver, thus allowing the patient to exhale a volume greater than his/her usual forced vital capacity (FVC). There are many different devices on the market designed to provide PEP therapy. Some combine PEP with high frequency oscillations of the airflow, the therapy being defined in this case as Oscillatory PEP (OPEP). Among the OPEP devices we mention Flutter, Acapella, RC-Cornet, Lung Flute and the PEP Bottle. All these devices are characterized by breathing against intermittent expiratory resistance to induce oscillations of variable frequency (depending on the device or use), which are transmitted to the respiratory tract during the expiratory cycle. The PEP component encourages the flow of air behind the secretions, while the oscillation induces vibrations within the bronchial walls to move the secretions into the lumen, and the repeated accelerations of the expiratory flow facilitate the movement of the secretions from the peripheral airways to the central ones (31). Studies investigating this strategy are very contradictory. In several long-term studies conducted on patients with Cystic Fibrosis, PEP was found to be more effective with respect to PD and percussion (32), the OPEP device Flutter (33), and high-frequency chest-wall oscillation (HFCWO) (34). However, a Cochrane review (5) showed no significant differences between PEP and other ACTs in individual treatments or in treatments with a duration of <3 months, in terms of FEV1. A long-term study found no significant difference in outcome between ACBT, AD, PEP, Flutter and RC-Cornet (25). In a 2017 Cochrane review conducted by Lee et al. (35), nine studies were analyzed, involving a total of 213 patients with bronchiectasis. From the comparison between PEP therapy and other clearance techniques (ACBT, ELTGOL, AD, and PD), it was concluded that there are no significant differences between them in terms of health-related quality of life (HRQOL), dyspnoea, mucus expectoration or lung volumes.

### Mechanical devices

#### Cough assist (or Mechanical Insufflator/Exsufflator)

The Mechanical Insufflator/Exsufflator (I/E) is a device that produces changes in the airflow inside the bronchial tree in such a way as to vicariate the cough (36, 37). It is used mainly in patients with neuromuscular pathologies or respiratory muscle deficiency, who have a hypo valid or ineffective cough. An ineffective cough causes retained secretions, chronic inflammation and infections, increased airway resistance, decreased lung compliance and respiratory failure (38). Even a single treatment would lead to a short-term reduction of dyspnoea, as demonstrated in patients with Duchenne Muscular Dystrophy (39). Several studies have analyzed the peak of cough (PCEF), and demonstrated with the use of mechanical I/E an increase in PCEF, and therefore an increase in the effectiveness of the cough, particularly in patients with neuromuscular diseases (40–42). Vianello et al. (43) in 2005 demonstrated its effectiveness in preventing intubation, while in 2009 Chatwin and Simonds (44) stated that its use decreases the treatment time in patients with neuromuscular disease (44). There are precise guidelines on the use of cough assist treatment in patients with NMD (45), while it has not been found to be very effective in patients with COPD (46).

#### Percussive Intrapulmonary Ventilation

Percussive Intrapulmonary Ventilation (IPV) is a device developed in 1979 by Forrest Bird. It consists of a high-pressure flow generator and a valve for stopping the flow. While the patient is breathing normally, the device provides the patient with high-frequency mini bursts of air (50–550 cycles per minute), thus creating an internal vibration (or percussion) in the lung. The vibration should promote clearance (47). The IPV device can also provide ventilatory support in patients with neuromuscular disease (48) and in patients with COPD (49). There are conflicting opinions, however, about IPV in the literature. Among the positive reports, IPV was found to improve the clearance of secretions in patients with Duchenne Muscular Dystrophy compared to conventional physiotherapy (FET and manually assisted cough) (50). And in 2006 Clini et al. (51) showed that the combination of conventional physiotherapy with IPV, compared to conventional physiotherapy alone, improved the PaO_2_/FiO_2_ ratio and maximum expiratory pressure (MEP) and reduced the incidence of pneumonia. Newhouse et al. (52) in 1998 compared the use of IPV, Flutter and PD with Percussion and HFCWC in patients with Cystic Fibrosis and found that they all had the same efficacy. Paneroni et al. (53) in 2011 compared IPV with conventional physiotherapy (combination of FET, PD, Percussion and Vibrations) and did not find any difference, except for a lower level of dyspnoea in patients treated with IPV. A systematic review conducted by Reychler et al. (54) investigated the effects of IPV use in patients with COPD and Cystic Fibrosis and concluded that the technique is not supported by strong enough evidence to recommend its use on a daily basis. However, it may offer some benefit during COPD exacerbations by improving gas exchange and possibly reducing hospitalization days.

#### High-Frequency Chest-Wall Oscillation (HFCWO)

High-frequency chest-wall oscillation (HFCWO) or high-frequency chest-wall compression (HFCWC) is a strategy for secretion clearance based on use of an inflatable vest connected to a compressor that can determine rapid inflation and deflation of the vest. This creates oscillations with a frequency of 5–25 Hz, which are transmitted through the chest wall to the entire bronchial tree. The oscillations should increase the interaction between the airflow and the mucus, increasing the cutting forces and thus decreasing the viscoelasticity of the secretions (55). Furthermore, according to Chang et al. (56), the oscillations should improve ciliary activity. There are conflicting studies on this strategy in the literature. The first studies carried out showed a significant increase in the expectoration of secretions compared to baseline and control, but no difference compared to other techniques (DP, Percussion and PEP) (57). Allan et al. (58) in 2003 reported that the potential benefits of HFCWO could be obtained in various clinical conditions (asthma, COPD, neuromuscular diseases, post-operative patients). However, more recent studies have suggested that this strategy eliminates a smaller amount of secretions than other unblocking techniques during an infectious exacerbation (59), and that it may even increase the frequency of exacerbations compared to PEP therapy (34). Nicolini et al. (60) compared a large variety of unblocking techniques, including HFCWO, to a control group that did not receive any type of intervention. All the techniques, including HFCWO, achieved better results in all outcomes than the control, but it was not possible to affirm the superiority of one technique over another.

#### Uniko - TPEP®

Uniko - TPEP® is a new generation temporary positive expiratory pressure (TPEP) device. At the beginning of the patient's expiratory phase, this device delivers a pulsed flow (about 42 Hz) contrary to the exhaled air resulting in a very low positive pressure of about 1 cmH_2_O. This pulsed flow stops before the end of the exhalation, so that the end of the expiratory phase is spontaneous, without any pressure support. The flow delivered is so slight that it does not create an excessive workload for the patient. The interruption of resistance at the end of expiration causes a pressure gradient, which can help reduce pressure within the airways thus improving the elasticity of the lung walls and consequently causing a reduction in excessive overdistention. The vibration generated by the pulsed flow is transmitted throughout the respiratory tract, where its effect is to detach the secretions from the internal walls of the lungs. The shape of the mouthpiece is such that the patient has to carry out an active non-forced exhalation. The resulting “open glottal exhalation,” in addition to prolonging the expiratory phase, produces an acceleration of the expiratory flow. This makes Uniko-TPEP® a very useful device for the drainage of secretions and reducing air-trapping. Although the device has been on the market for a relatively short time, there are already several studies published in the literature. D'Abrosca et al. (61) compared PEP therapy with TPEP therapy (delivered with the Uniko device). Physiological parameters of both groups improved significantly and in a similar way. A subgroup analysis suggested that TPEP could provide greater benefit to patients with emphysema or on oxygen therapy, while PEP therapy would be of more benefit for patients on mechanical ventilation. Nicolini et al. (62) compared intermittent positive pressure breathing (IPPB) with TPEP and showed that the two techniques both significantly improve dyspnoea, quality of life and lung function in patients with severe COPD, although IPPB would seem to be more effective. Again, Nicolini et al. (63) compared TPEP with OPEP, in patients with severe COPD, and found that both techniques are useful for the treatment of COPD but only TPEP reduces exacerbations. Other studies also show that patients with severe COPD treated with TPEP have a lower incidence of exacerbations, as TPEP improves respiratory function parameters, improving dyspnoea (64). A randomized multicentre trial (65) concluded that TPEP improves lung volumes and accelerates the improvement of bronchial dimensions in patients with lung disease and hypersecretion.

#### Vacuum Technique

There is one device available that employs the vacuum technique to clear the airways: Free Aspire®. The device uses Expiratory Flow Accelerator (EFA®) technology, which accelerates the expiratory flow, promoting deep drainage and secretions removal in patients with or without ineffective cough, without applying any pressure in the airways. The secretions safely reach the upper airways where the patient can expel or ingest them in a physiological way through the mechanism of the mucociliary system.

The acceleration of expiratory flow is produced by a Venturi effect and the amount of flow acceleration is proportional to the expired airflow. The movement of air over the mucus layer develops a cutting force on the surface itself. When the shear force exceeds the surface tension in the mucous layer, the mucus begins to move in the direction of the airflow and secretions are “dragged” from peripheral to central areas. Bertelli et al. (66) described the clinical case of a 3-year-old girl with type-1 spinal muscular atrophy (SMA), and claimed that, in patients with ineffective cough and, hence, compromised secretions removal, Free Aspire is a safe and effective machine for the removal of bronchial secretions. Garuti et al. (67) described the use of Free Aspire in eight children with infantile cerebral palsy (a disease that, due to deformities of the ribcage, reduces cough and, given the patient's inability to collaborate, makes secretions management very problematic). They concluded that Free Aspire is safe and effective in reducing the impact of respiratory exacerbations in terms of visits to the pediatrician, days spent in hospital and days of antibiotic therapy. They also claimed that regular use of the device maintains these effects over time. Our group recently demonstrated the short-term efficacy of Free Aspire in tracheostomised patients; the device reduced the number of daily and deep aspirations in patients with bronchial dimensions from the first day of treatment (68). Likewise, Patrizio et al. (69), found a significant pre-post- improvement in arterial blood gases (ABG), and a significantly greater improvement with Free Aspire than Bubble PEP in PCEF, maximum inspiratory pressure (MIP), and the 6-min walk test (6MWT) post-treatment in patients with stable severe COPD.

#### Non-invasive Ventilation

NIV can be used to improve lung volume (PIP) associating the PEP effect during the expiratory phase. This option is used for patients who are not able to perform other ACTs without assistance, especially with severe/end-stage disease, inspiratory muscle weakness, severe hypoxia and dyspnoea. It can be useful adjuncts to other ACTs especially when people have difficulty expectorating (70–74).

Literature offers few studies demonstrating the effectiveness of this technique; most studies focus on cystic fibrosis patients and patients in acute settings.

It can certainly be considered a valid tool to assist traditional techniques.

#### Combined ACTs

In the last few years, new devices have begun to emerge that combine different techniques. The aim of these is to improve the clearance of excessive secretions in patients whose airway obstruction is driven by more than one mechanism. As an example, there is a device is now combining the vacuum with I/E techniques. It was designed to potentiate clearance by applying two different ACTs acting with different mechanisms; the principle is analogous to that of combining two different drugs in an inhalation device to treat asthma or COPD. Further research is needed (though to perform this adequately will not be easy) to investigate the effects of these new devices in different categories of patients.

#### COVID Patients

Clearance procedures should be administered only when considered strictly needed for the clinical improvement of the patient (75).

Assessment of mucus encumbrance or expectoration difficulties should be considered in all patient reporting pre-existing hyper-secretive condition, those after extubation or weaning from mechanicalventilation, those reporting phlegm or sticky mucus and productive cough. Early reports indicate that patients with COVID-19 do not show airway mucus hypersecretion, however, patients with specific comorbidities (e.g., COPD, cystic fibrosis, neuromuscular disease) might actually need respiratory support due to airway secretion retention or ineffective cough. In case of clinical signs for presence of airway secretion (by hearing, feeling, or chest x-ray), different techniques and devices can be applied to mobilization or evacuation (76). In hypersecretive patients, the use of continuous or temporary positive expiratory pressure device with or without oscillation (PEP, TPEP, OPEP), should be considered, alone or in combination with lung expansion strategies, to enhance lung volume recruitment, to better control the expiration flow and to facilitate peripheral and proximal mucus mobilization. Flow-dependent low resistance positive expiratory pressure (PEP) systems, with an antibacterial filter on expiration circuit, are more tolerated and should be preferred to high resistance and threshold-PEP, mostly in weaker or symptomatic patients

Since cough is one of the most annoying symptoms in COVID-19 lung involvement and can cause dyspnea or chest pain, forced expiratory flows (huffs) should be preferred to expectorate. Among ACTs, those that enable patient to auto-treatment should be preferred (77).

## Conclusions

Based on the currently available ACTs, on the literature and on our experience, we have devised an algorithm to help in the decision-making as to what is the right ACT to use for each patient in our clinical practice.

We acknowledge that this schematic representation of the decision-tree for ATCs is somewhat arbitrary and reflects in many cases our own clinical judgement more than evidence-based data from the literature. Nevertheless, we propose it as a tool to help others confronted with the clinical problem of patients who require an ACT and the need to decide which one is best. We hope it may help in their decision-making and in building their clinical experience. Our goal, of course, is to improve the outcomes for our patients.

## Author Contributions

SB drafted the paper. BB reviewed it and made important changes. All authors reviewed the literature and read the manuscript and made comments.

## Conflict of Interest

The authors declare that the research was conducted in the absence of any commercial or financial relationships that could be construed as a potential conflict of interest.
